# Editorial: Immune interactions with pathogenic and commensal fungi

**DOI:** 10.3389/fimmu.2022.1108022

**Published:** 2023-01-04

**Authors:** Wenjie Fang, Carolina Coelho, Cunwei Cao, Lei Zhang, Wanqing Liao

**Affiliations:** ^1^ Shanghai Key Laboratory of Molecular Medical Mycology, Department of Dermatology, Second Affiliated Hospital of Naval Medical University, Shanghai, China; ^2^ Medical Research Council (MRC) Centre for Medical Mycology at University of Exeter, Exeter, United Kingdom; ^3^ Department of Dermatology and Venerology, First Affiliated Hospital, Guangxi Medical University, Nanning, China; ^4^ Department of Dermatology, The third affiliated hospital of Xi’an Jiaotong University, Shaanxi Provincial People’s Hospital, Xi’an, China

**Keywords:** antifungal immunity, cytokines, candidiasis, cryptococcosis, aspergillosis

The fungal kingdom encompasses symbiotic environmental organisms critical to functioning of our ecosystem; fungi living in close relationships with humans can be commensal and, unfortunately, opportunistic pathogens and fungi that explicitly cause diseases (1). Human diseases caused by fungi are an increasing health problem resulting in 149 million cases and 1.7 million deaths globally every year. The WHO recently released a list of fungal “priority pathogens,” which classified 19 clinical important fungi into Critical (*Cryptococcus neoformans*, *Candida auris*, *Aspergillus fumigatus*, and *Candida albicans*), High Priority (*Candida glabrata*, *Histoplasma* spp., eumycetoma causative agents, *Mucorales*, *Fusarium* spp., *Candida tropicalis*, and *Candida parapsilosis*), and Medium Priority (*Scedosporium* spp., *Lomentospora prolificans*, *Coccidioides* spp., *Candida krusei*, *Cryptococcus gattii*, *Talaromyces marneffei*, *Pneumocystis jirovecii*, and *Paracoccidioides* spp.). These fungi cause diseases with varying degrees of severity in individuals with normal or impaired immunity. Fungal diseases are a key illustration that disease states result from a complex interaction between pathogens and the host immunity. In addition, the role of the fungal microbiome on immunity and disease is an emerging area of interest and many underlying mechanistic processes remain elusive. Herein, a better understanding of the molecular and cellular basis of antifungal and fungal microbiome immunity will undoubtedly provide opportunities for novel therapeutic strategies and applications.

In this Research Topic, we collect 16 articles involving 102 authors, focusing on the underlying interaction mechanisms of commensal and pathogenic fungal organisms with the host.

Neutrophils are essential components of the host innate immunity against fungal infections. In addition to phagocytosis, degranulation, and reactive oxygen species production, the formation of neutrophil extracellular traps (NETs) is an important mechanism of neutrophil action against all pathogens (2). Zhong et al. and He et al. describe the formation, induction, and function of NETs and also summarize the effects of NETs on deadly fungi such as *Aspergillus fumigatus*, *Cryptococcus neoformans*, and *Candida albicans.*

The adrenal gland is a key element of the Stress Response System that may be affected by pathogenic microorganisms. Using single-cell RNA sequencing technology, Zhang et al. explore the complex adrenal microenvironment and how this is affected during candidemia in a murine model. The single‐cell transcriptomic analysis reveals increased immune-adrenal interactions, proliferated endothelial cells, and immune cell infiltration in adrenal glands.

Vulvovaginal candidiasis (VVC) is a vaginal infection caused by either *C. albicans* or non-*albicans Candida* (NAC), affecting virtually all women at least once during their lifetimes. Recurrent vulvovaginal candidiasis (RVVC) is a particular condition in which VVC patients experience four or more episodes of infection per year. The immunological aspects of VVC remain largely unknown. Ge et al. follow 98 VVC patients and found lower interferon γ (IFN-γ), tumor necrosis factor (TNF), and interleukin 17F (IL-17F), as well as higher interleukin 4 (IL-4), interleukin 6 (IL-6), and interleukin 10 (IL-10) levels in serum of RVVC than VVC patients. This study indicates that the T helper type 1/2 (Th1/2) balance could be involved in recurrent VVC. The local innate immune state of the vaginal mucosa epithelium is of great importance in the recognition and elimination of invading fungal pathogens. Zhang et al. explore the role of the epidermal growth factor receptor (EGFR)-mitogen-activated protein kinase (MAPK) signaling pathway in VVC pathogenesis and highlight the remarkable immunogenic differences between *C. albicans* and NAC species in host–microbe interactions. Using an animal model, Guo et al. prove that boric acid gel effectively controls symptoms of VVC, likely by upregulating Th1 and Th17 cytokines and inhibiting Th2 cytokines.

Chronic mucocutaneous candidiasis (CMC) is characterized by recurrent or persistent infections by *Candida* spp. on the skin, nails, and mucous membranes, and signal transducer and activator of transcription 1 (STAT1) gain-of-function (GOF) mutations are responsible for more than half of congenital CMC cases (3). Lu et al. report on a CMC patient with STAT1 GOF (c.Y289C) mutation and the changes in immune cell populations. Single-cell RNA-seq analysis confirms the defects with alteration in genes related to antigen presentation and antimicrobial functions. This study establishes the feasibility of single-cell RNA-seq technology as a strategy for investigating detailed immune pathogenic responses, which will further allow a deeper understanding of CMC.

Cryptococcosis, caused by *Cryptococcus neoformans/Cryptococcus gattii* complex species, is a systemic mycosis, which can manifest *via* cryptococcal meningitis, pneumonia, and blood or skin infections. In a study by Yang et al. featuring both inhalational and intravenous mouse models, the *csn1201Δ* strain was shown to decrease tolerance to various stressors *in vitro*, as predicted, indicating that *CSN1201* may promote the exposure of cell wall components and thus induce a protective immune response. These results forecast that the *CSN1201* deletion significantly blocks the pulmonary infection and extrapulmonary dissemination of *C. neoformans*, supporting the importance of cryptococcal *CSN1201* in pulmonary immune responses and disseminated infection. Jiang et al. study the correlation between cerebrospinal fluid (CSF) immune response and disease severity by following 128 cryptococcal meningitis (CM) and 30 pulmonary cryptococcosis among HIV-negative individuals. This research indicates that both CSF pro- and anti-inflammatory cytokines and chemokines are elevated in CM, and prognostic analysis shows its association with disease severity.

*Aspergillus* spp., as one of the most prevalent opportunistic fungal pathogens, causes a wide range of pulmonary infections or allergic responses in humans, including invasive pulmonary aspergillosis, chronic pulmonary aspergillosis, and allergic bronchopulmonary aspergillosis (4). Cai et al. discuss the effects of microbiomes on pulmonary aspergillosis (PA) and highlight that lung and gut microbiomes can prevent PA by affecting the growth of *Aspergillus* spp. or host immunity. This review provides a novel aspect for PA treatment from a microbiome perspective.

Mutations in the caspase recruitment domain family member 9 (CARD9) gene are known to cause immune disorders and are a major risk factor for mycoses (5). Huang et al. isolate *Phialophora expanda* from a patient with chromoblastomycosis due to a CARD9 mutation. Compared with a previous case infected by *P. americana*, the patient infected with *P. expanda* shows stronger local immune responses. Song et al. report a patient with CARD9 deficiency who is reinfected by *P. verrucosa* with a period of 10 years apart. By barcoding gene sequencing and Coomassie-stained whole-protein analysis, Song et al. predict that the two isolates belong to one strain, and they further find an upgrading trend of lysine lactylation in the two isolates through a 10-year period.

Human-disseminated protothecosis is a rare infection associated with debilitated hosts. With the increasing numbers of immunocompromised individuals throughout the world, protothecosis is suspected to be underestimated and misdiagnosed due to the lack of specificity of clinical features and low awareness among clinicians. Wang et al. summarize the etiology, epidemiology, clinical aspects, diagnostic characteristics, and treatment of disseminated protothecosis to better understand this emerging infection. This fungus is ubiquitous in natural environments, colonizes the skin, and shows resistance to multiple antimicrobial agents, and thus *Prototheca* spp. may potentially be dangerous to susceptible populations. Wang et al. confirm *P. wickerhamii* and *P. zopfii* to be the dominant species that cause disseminated infections. This study also confirms that disseminated protothecosis is most frequently found in the skin and could be an important diagnostic sign for this disseminated disease.

The antifungal immunity associated with invasive mucormycosis is still unclear. *Lichtheimia corymbifera* is the most often isolated pathogens of mucormycosis. Montaño et al. study the host immune response of human monocytes to *L. corymbifera* and to show that the Toll-like receptor 4/nuclear factor-kappa B (TLR-NF-kB) axis is likely involved.

Cutaneous disseminated sporotrichosis is a rare infectious condition, occurring mostly in immunocompromised patients. Zhuang et al. report a case of refractory cutaneous disseminated sporotrichosis in an immunocompetent individual. The patient resisted multiple antifungal treatments and was eventually treated by oral solution of potassium iodide. While few cases exist to support the wider use of this therapy, potassium iodide may be an option for multidrug-resistant *Sporothrix* infection.

In conclusion, the publications collected in this Research Topic of “Immune Interactions with Pathogenic and Commensal Fungi” reflect the variety of responses during antifungal immunity against a wide range of clinical important fungal pathogens. We hope this collection will deepen our understanding of diseases caused by human fungal pathogens. Moreover, we suggest a need to strengthen the scientific investment in immune pathogenesis research in mycosis because of the high mortality worldwide, especially in immunocompromised individuals such as HIV patients. The limited number of current antifungal therapies, the emergence of fungal isolates with genetically encoded resistance, and the relatively high toxicity call for a better understanding of immune interactions with fungi in order to contribute to novel strategies for combatting fatal human fungal diseases.

## Author contributions

All the authors have made extensive, direct, and intellectual contributions to the present work and approved it for publication.

## References

[1] Underhill DM Pearlman E . Immune interactions with pathogenic and commensal fungi: A two-way Street. Immunity (2015) 43(5):845–58. doi: 10.1016/j.immuni.2015.10.023 PMC4865256 26588778

[2] Brinkmann V Reichard U Goosmann C Fauler B Uhlemann Y Weiss DS . Neutrophil extracellular traps kill bacteria. Sci (New York NY). (2004) 303(5663):1532–5. doi: 10.1126/science.1092385 15001782

[3] Kirkpatrick CH . Chronic mucocutaneous candidiasis. Pediatr Infect Dis J (2001) 20(2):197–206. doi: 10.1097/00006454-200102000-00017 11224843

[4] Kolwijck E van de Veerdonk FL . The potential impact of the pulmonary microbiome on immunopathogenesis of aspergillus-related lung disease. Eur J Immunol (2014) 44(11):3156–65. doi: 10.1002/eji.201344404 25256637

[5] Wang X Wang W Lin Z Wang X Li T Yu J . CARD9 mutations linked to subcutaneous phaeohyphomycosis and TH17 cell deficiencies. J Allergy Clin Immunol (2014) 133(3):905–8.e3. doi: 10.1016/j.jaci.2013.09.033 24231284

